# A Decade of NT-proBNP in Acute Kawasaki Disease, from Physiological Response to Clinical Relevance

**DOI:** 10.3390/children5100141

**Published:** 2018-10-12

**Authors:** Audrey Dionne, Nagib Dahdah

**Affiliations:** 1 Department of Cardiology, Boston Children’s Hospital, Department of Pediatrics, Harvard Medical School, Boston, MA 02115, USA; Audrey.Dionne@cardio.chboston.org; 2 Department of cardiology, CHU Sainte-Justine, Montreal University, 3175, Cote Sainte-Catherine, Montréal, QC H3T 1C5, Canada

**Keywords:** Kawasaki disease, biomarker, predictive value, child, heart, inflammation

## Abstract

Kawasaki disease (KD) is an inflammatory febrile illness of early childhood and the primary cause of acquired heart disease during childhood. Coronary artery aneurysms (CAA) are a serious complication of KD, leading to ischemic heart disease, myocardial infarction, and sudden cardiac death. Timely diagnosis in the first ten days of fever is crucial to reduce the risk of coronary artery complications. Nitrogen-terminal B-type natriuretic peptide (NT-proBNP), originally used for the management of adults with heart disease, was shown to be useful in the diagnosis and management of patients with KD. NT-proBNP is released by cardiomyocytes in response to mechanical factors such as the dilation of cardiac chambers, and to pro-inflammatory cytokines. The utility of NT-proBNP as a biological marker in KD is based on the universal myocardial inflammatory component early in the course of the disease. Patients with KD have higher NT-proBNP at the time of diagnosis than febrile controls, with a pooled sensitivity of 89% (95% confidence interval 78–95), and a specificity of 72% (95% confidence interval 58–82). The positive likelihood ratio is 3.2:1 (95% confidence interval 2.1–4.8). Moreover, patients with resistance to intravenous immunoglobulin treatment and CAA were found to have higher levels of NT-proBNP, suggesting a prognostic role. Nevertheless, the non-specificity of NT-proBNP to KD limits its use as a stand-alone test. In this light, a tentative associative retrospective diagnostic algorithm was highly reliable for including all cases at risk of CAA, which warrants further prospective studies for a better diagnostic index of suspicion and risk stratification of patients.

## 1. Introduction

Five decades after the initial description of Kawasaki disease (KD), this inflammatory febrile illness of early childhood earned deserved worldwide interest on clinical and the research grounds [1]. The interest established in KD is consequential to the deleterious coronary artery aneurysm (CAA) complication, which represents the principal cause of ischemic heart disease and myocardial infarction early in life [1,2,3]. An accurate and timely diagnosis of the disease is a fundamental requirement to secure timely treatment with immunoglobulins and curtail the risk of developing CAA. The diagnosis of KD remains difficult, especially in the absence of a gold standard and an operator-independent biomarker. From the principle that the solution to a riddle always lies within it, investigating information expressed by the heart during acute KD proved fruitful on many levels. Here, we review the role of myocardial immune inflammation in KD and the subsequent myocardial release of B-type natriuretic peptide (BNP) and its biologically inert Nitrogen-terminal pro B-type natriuretic peptide (NT-proBNP), as reported during the last decade, and how it bears a significant role in increasing the level of diagnostic accuracy of acute KD.

## 2. NT-proBNP

The value of serum NT-proBNP in acute KD is based on the universal myocardial component from the histological and functional perspectives [4]. Myocarditis is the earliest feature of KD and is present in the first ten days of illness, compared to coronary vasculitis and aneurysms which usually develop after ten days from the onset of fever [5,6]. Moreover, myocarditis, at least subclinical, is present in virtually all patients, regardless of the presence or absence of CAA. Evidence of myocarditis in the early phase of KD has been shown on post-mortem specimens [6], cardiac biopsies [7], nuclear imaging [8,9], and echocardiography [10,11]. Cardiac biopsies performed in 201 patients between 1 month and 11 years after the diagnosis of KD showed varying degrees of cellular infiltration, fibrosis and abnormal myocardial structure in 100% of patients regardless of the presence or absence of significant coronary artery injury [7]. Nuclear imaging with either gallium-67 or Tc-99 HMPAO-labeled white blood cells showed evidence of myocarditis in approximately 60% to 80% of patients studied 5 to 16 days into fever [8,9]. Echocardiograms in the acute phase have shown a depressed left ventricular systolic function on initial assessment [10,11]. Moreover, the left ventricular ejection fraction correlated with systemic evidence of inflammation [10]. Thus, the omnipresence of myocarditis in the early stage of the disease provides a fertile ground for diagnostic and prognostic biomarkers.

BNP and NT-proBNP are currently used in the diagnosis and management of heart disease in adults. NT-proBNP is a physiologically inactive fragment of BNP. It is released when the active hormone is cleaved from proBNP, its precursor. Synthesis of BNP in the cardiomyocytes is controlled by mechanical factors, such as the dilation of cardiac chambers, and neurohormonal factors [12,13,14]. Unlike cardiac troponin, the main relevance of BNP release in certain cardiac stressful situations is its reliability preceding the manifestation of myocardial ischemia or infarction.

In addition to mechanical factors, pro-inflammatory cytokines such as tumor necrosis factor alpha or interleukin-1 beta, which are known to be increased in the acute phase of KD, induce the secretion of BNP from myocytes at the transcriptional and translational levels [15,16,17]. Moreover, prior studies have found a linear relationship between NT-proBNP and inflammatory markers in the acute phase of KD, including an increase in C-reactive protein (CRP) and a decrease in the serum albumin level [17,18].

NT-proBNP proved superior to BNP in the evaluation of KD at the onset of the disease, thanks to the longer half-life of the former providing greater sensitivity and specificity [19,20,21]. Pre-proBNP, the precursor of the active peptide, is a 134 amino acid molecule. Following its secretion by the myocardium, it undergoes cleavage into a 26 amino acid signal peptide and proBNP, a 108 amino acid peptide. Pro-BNP then separates as BNP, the active ring segment, and the metabolically inactive N-terminal fragment of proBNP, NT-proBNP. BNP, the active component of the molecule, is more readily metabolized by the kidney, where it is primarily eliminated, and the vascular tree. It has a short half-life of 20 min. The inactive moiety, NT-proBNP, circulates unchanged in the serum for a longer period of time (60–120 min) and is similarly cleared primarily by the kidney [20,21]. From a mechanistic point of view, the answer lies in the observed low serum Na in acute KD compared to other febrile illnesses. In our series, hyponatremia (Na <135 mEq/L) was present in 50% of cases [4], and between 26% and 69% in other series [5]. The metabolic role of natriuretic peptides enhances sodium elimination by the kidney as a natural diuretic. In fact, our series of 117 patients showed the fractional excretion of sodium to increase at the onset of KD, with a secondary reduction in the plasma concentration of Na, all proportional to serum NT-proBNP levels [5]. A lower serum sodium concentration, concomitant with lower urine specific gravity, contradicts the theory of an increased antidiuretic hormone activity where an elevated specific gravity is expected.

The interpretation of NT-proBNP levels in children is complicated by the fact that the serum level varies with age [22,23]. NT-proBNP levels are very high soon after birth, decrease drastically in the first days of life, and then decrease gradually with age throughout early childhood. A study on NT-proBNP by McNeal-Davidson et al. evaluated different definitions of elevated NT-proBNP (receiver operating characteristics (ROC) analysis, upper limit for age, and Z-score for age). The upper limit for age and the Z-score for age were superior in the ROC analysis for sensitivity, specificity, and predictive values compared to un-normalized raw values [4]. A more recent study on 149 KD patients and 506 controls determined that a Z-score > 2 for age was the optimal cut-off value, yielding a sensitivity of 47% and a specificity of 98% [24]. The variation in the cut-off values to define normal and elevated NT-proBNP explains the variation in the performance of NT-proBNP in the diagnosis of KD across studies. Prospective studies evaluating the role of NT-proBNP in the diagnosis of KD had cut-off values of between 98 and 260 pg/mL with a sensitivity varying between 66% and 98%, accordingly [25].

## 3. Value of Nt-ProBNP in the Diagnosis of Kawasaki Disease

Since the first report on NT-proBNP in KD in 2006, multiple studies have evaluated this potential diagnostic marker [4,24,25,26,27]. All the studies found higher NT-proBNP values for patients with KD compared to febrile controls. A recent meta-analysis including 428 patients and 709 controls confirmed those findings, with a pooled sensitivity of 89% (95% confidence interval 78–95), a pooled specificity of 72% (95% confidence interval 58–82), and a positive likelihood ratio of 3.20:1 (95% confidence interval 2.10–4.80) [25]. Those results were found in patients with complete and incomplete KD clinical criteria.

However, as stated in the meta-analysis, while NT-proBNP is useful in the evaluation of patients with KD, its positive likelihood ratio of 3.20:1 [95% confidence interval 2.10–4.80] is not sufficient for use as a stand-alone test [25]. We recently retrospectively studied the value of NT-proBNP in a diagnostic algorithm to identify patients with complete and incomplete KD clinical criteria. In addition to the NT-proBNP age-calculated Z-score (>2.0), the algorithm is based on three simple tests: echocardiographic coronary artery Z-score (>2.5) at onset; elevated CRP (>10.0 mg/L); and/or low serum albumin (<30 g/dL) [28]. Applying the proposed algorithm to a retrospective series of 124 patients would have identified 97% of them and directed them to therapy. According to the algorithm, the positive diagnosis of KD was based on a high serum NT-proBNP Z-score alone in 64%. The remaining assertion of the diagnosis of KD was aided by the diagnosis of coronary dilatation at onset in an additional 11% and on a high serum CRP level and/or low serum albumin concentration in the remaining 22% of these patients. In addition, all the patients who had developed coronary aneurysms would have been diagnosed and referred for therapy using the algorithm. This NT-proBNP based algorithm was also superior to the 2004 American Heart Association algorithm to identify and refer for treatment patients with incomplete KD clinical criteria in our cohort (95% versus 45%, *p* < 0.001) [28]. However, very few studies have explored the value of NT-proBNP as part of a diagnostic algorithm, or in combination with clinical criteria and laboratory values.

## 4. Nt-ProBNP and Response to Intravenous Immunoglobulin (IVIG) Treatment

Persistent or recurrent fever after intravenous immunoglobulin (IVIG) treatment is one of the strongest risk factors for the development of CAA. In Japan, risk score systems are used to predict patients at high risk of IVIG resistance, and initial therapy is intensified in those patients [29,30,31]. However, those risk scores did not prove useful in other populations [32,33], and no single marker has been proven to predict resistance to treatment. NT-proBNP has been shown in some, but not all studies to predict or accompany resistance to IVIG treatment [34,35,36,37]. A large multicenter study showed that IVIG resistant patients had higher levels of NT-proBNP compared to non-resistant patients (1574 ± 3166 vs. 941 ± 2326 pg/mL, *p* < 0.001). In the study, the statistical significance remained relevant on a multivariate analysis for NT-proBNP, but also for polymorphonuclear white blood cells, for C reactive protein, and for aspartate aminotransferase and alanine aminotransferase [36]. Cut-off values between 629–1300 pg/mL have been described, with a sensitivity and specificity of 70–79% and 58–77% respectively [36,37]. The cut-off values to predict resistance to treatment are significantly higher than those used to diagnose KD.

## 5. Nt-ProBNP and Myocardial Function

Patients with elevated NT-proBNP had a significantly lower fractional shortening Z-score value at onset than those with normal NT-proBNP (−1.77 ± 1.94 and −0.81 ± 1.31, *p* = 0.014) [25]. While the left ventricular shortening fraction improved during convalescence, it remained significantly lower in patients with elevated NT-proBNP (0.122 ± 1.83 vs. 0.98 ± 1.75, *p* < 0.001) [35,38]. However, a recent study did not find an association between NT-proBNP and myocardial strain on 3-D speckle tracking imaging [39].

## 6. NT-proBNP and Coronary Artery Lesions

In a prospective study including 109 patients, patients with elevated NT-proBNP had a significantly higher rate of coronary artery dilation (defined as a Z-score ≥ 2.5) at the time of diagnosis (22% vs. 6%, *p* = 0.03) in a series where the rate of coronary artery dilation regressed at a 2–3 month follow-up (with an odds ratio of remaining dilatation of 1.28 (95% confidence interval 0.23–8.30) [38]. Similarly to IVIG resistance, while NT-proBNP Z-score for age was associated with coronary artery dilation, the diagnostic cut-off value of 190 pg/mL used for diagnosis of KD was not predictive of coronary involvement [25,40]. Instead, studies have reported cut-off values between 515–1300 pg/mL to predict coronary artery dilation, with a sensitivity and specificity of 73–95% and 61–85%, respectively [17,36,37,40].

## 7. Conclusions

NT-proBNP can be useful in the diagnosis of Kawasaki disease, although its sensitivity and specificity does not allow its use as a stand-alone test. NT-proBNP can be a useful prognostic marker in patients with KD. Higher levels of NT-proBNP are associated with resistance to IVIG treatment and CAA. Further prospective studies should investigate the value of NT-proBNP in an algorithm or in combination with other clinical criteria and laboratory values, which will likely increase its sensitivity and make it more valuable in the evaluation and risk stratification of KD patients.
